# Discovery of Novel Tankyrase Inhibitors through Molecular Docking-Based Virtual Screening and Molecular Dynamics Simulation Studies

**DOI:** 10.3390/molecules25143171

**Published:** 2020-07-11

**Authors:** Vladimir P. Berishvili, Alexander N. Kuimov, Andrew E. Voronkov, Eugene V. Radchenko, Pradeep Kumar, Yahya E. Choonara, Viness Pillay, Ahmed Kamal, Vladimir A. Palyulin

**Affiliations:** 1 Department of Chemistry, Lomonosov Moscow State University, 119991 Moscow, Russia; vladimber@qsar.chem.msu.ru (V.P.B.); av@digitalbiopharm.com (A.E.V.); genie@qsar.chem.msu.ru (E.V.R.); 2 A.N. Belozersky Institute of Physico-Chemical Biology, Lomonosov Moscow State University, 119991 Moscow, Russia; kuimov@genebee.msu.ru; 3 Digital BioPharm Ltd., Hovseterveien 42 A, H0301, 0768 Oslo, Norway; 4 Wits Advanced Drug Delivery Platform Research Unit, Department of Pharmacy and Pharmacology, School of Therapeutic Sciences, Faculty of Health Sciences, University of the Witwatersrand, Johannesburg, 7 York Road, Parktown 2193, South Africa; pradeep.kumar@wits.ac.za (P.K.); yahya.choonara@wits.ac.za (Y.E.C.); Viness.Pillay@wits.ac.za (V.P.); 5 School of Pharmaceutical Education and Research, Jamia Hamdard, New Delhi 110 062, India; pvc@jamiahamdard.ac.in

**Keywords:** tankyrase inhibitors, molecular docking, molecular dynamics, MM-PBSA, immunochemical assay, free energy perturbation

## Abstract

Tankyrase enzymes (TNKS), a core part of the canonical Wnt pathway, are a promising target in the search for potential anti-cancer agents. Although several hundreds of the TNKS inhibitors are currently known, identification of their novel chemotypes attracts considerable interest. In this study, the molecular docking and machine learning-based virtual screening techniques combined with the physico-chemical and ADMET (absorption, distribution, metabolism, excretion, toxicity) profile prediction and molecular dynamics simulations were applied to a subset of the ZINC database containing about 1.7 M commercially available compounds. Out of seven candidate compounds biologically evaluated in vitro for their inhibition of the TNKS2 enzyme using immunochemical assay, two compounds have shown a decent level of inhibitory activity with the IC_50_ values of less than 10 nM and 10 μM. Relatively simple scores based on molecular docking or MM-PBSA (molecular mechanics, Poisson-Boltzmann, surface area) methods proved unsuitable for predicting the effect of structural modification or for accurate ranking of the compounds based on their binding energies. On the other hand, the molecular dynamics simulations and Free Energy Perturbation (FEP) calculations allowed us to further decipher the structure-activity relationships and retrospectively analyze the docking-based virtual screening performance. This approach can be applied at the subsequent lead optimization stages.

## 1. Introduction

The tankyrase enzymes (TNKS1 and TNKS2, also known as PARP5a and PARP5b) belong to the poly(ADP-ribose) polymerase (PARP) superfamily. They play vital roles in mitosis control, telomere maintenance and regulation of the canonical Wnt pathway [1]. Since aberrant Wnt signaling is often associated with various cancers [2], TNKS enzymes are considered potential pharmacological targets for anti-tumor agents. The efforts aimed at using tankyrase inhibitors in combination with other drugs are also worth noting. It is believed that the synergistic effect could occur when tankyrase is inhibited simultaneously with cyclin-dependent kinases 4 and 6 [3] or phosphoinositide 3-kinase [4]. In addition, multitarget inhibitors able to act on the previously mentioned kinases and the TNKS enzymes could be beneficial thanks to diminished xenobiotic load.

Despite being a relatively new pharmacological target, TNKS enzymes have been extensively studied [5,6] and several hundreds of structurally diverse inhibitors have already been identified such as flavones [7], thiopyranopyrimidines (XAV939) [8], 1,2,4-triazoles [9] and 2-phenylquinazolinones [10], to name a few. For more comprehensive discussion of the currently known TNKS inhibitors the reader is referred to the reviews [11] and [12].

Nevertheless, novel inhibitor chemotypes are desirable since they could potentially lead to the discovery of highly selective inhibitors suitable for combination therapy or multitarget compounds. Taking this into account, we have chosen the molecular docking-based virtual screening as a primary tool to search for the TNKS inhibitors. To some extent, this choice was determined by our previous experience in the development of target-oriented scoring functions [13]. That study provided information about the optimal docking parameters able to enhance the virtual screening efficiency. The machine learning (ML) models implemented in that work could also serve as additional screening filter complementing standard scoring functions.

In order to prioritize the results of docking-based virtual screening or to optimize the structure of lead compounds, the binding free energies could be calculated. Although demanding greater computational efforts compared to docking, they usually provide better correlation of calculated binding energies with experimental values [14]. The methods for calculating the binding energies can be roughly classified into the so-called endpoint and pathway methods. Representatives of the first group are such popular approaches as MM-PBSA and MM-GBSA (molecular mechanics, Poisson-Boltzmann / Generalized Born, surface area) [14]. These methods explicitly take into account the solvation effect on binding energy using a continuum solvent model based on the Poisson-Boltzmann equation or the Generalized Born model. Thus, they could show better results than docking in terms of the ranking power. The second category includes the free energy perturbation (FEP) and thermodynamic integration (TI) methods [15,16]. They are substantially more computationally intensive compared to the endpoint methods, let alone docking; however, they tend to attain the best agreement with experimental values.

Considering the high computational complexity of these methods, presently they cannot be used for large-scale virtual screening. In this study, the endpoint (MM-PBSA) approach was employed during virtual screening and the pathway methods we used to retrospectively analyze the docking results. In addition, we have tried to determine the source of inconsistency between the docking and MM-PBSA predictions and the observed inhibitory activities.

This paper is organized as follows. First, we discuss virtual screening procedure and its main steps. Next, we provide results of biological evaluation of the selected compounds. Finally, the differences in the binding of discovered active compounds are discussed based on the results of molecular docking, molecular dynamics simulation and binding energy calculations.

## 2. Results and Discussion

### 2.1. Structure-Based Virtual Screening

The library of commercially available compounds for virtual screening of potential TNKS inhibitors was compiled on the basis of the ZINC open database [17] using its website interface and taking into account the following main considerations—(1) drug-likeness and lack of PAINS (pan-assay interference compounds) patterns and (2) availability for purchase in our region. The resulting library comprised more than 1.7 million compounds. The virtual screening workflow involved the following steps (protocol details are explained in the Materials and Methods section):

- Semi-rigid molecular docking into the tankyrase binding site was performed using the Smina software and the compounds were selected based on the values of the *Vinardo* scoring function. The previously developed machine learning-based scoring function was also employed as an additional screening filter.
- Compounds that have acceptable molecular weight, lipophilicity (LogP), aqueous solubility and human intestinal absorption as well as low risk of hERG-mediated cardiac toxicity were selected (the properties were predicted using previously developed QSPR/QSAR models).
- Expert analysis of the resulting compounds was performed to eliminate potentially unstable, reactive or excessively complex structures.
- For the seven selected compounds, molecular dynamics simulations and MM-PBSA calculations were carried out in order to provide additional independent assessment of their potential activity.
- Biological evaluation of inhibitory activity of the selected compounds was carried out.

Even with steady improvement in the accuracy of computational methods over the years, it is not uncommon when only a fraction of the compounds predicted to be active shows some real activity. To minimize these risks, we used consensus scoring including molecular docking, ML scoring, QSAR models for the physico-chemical profile prediction and MM-PBSA method for binding energy estimation.

Although the MM-PBSA binding energy estimates show a broad range of correlations to the experimental values [18], they are widely used in practice and could, in our opinion, provide useful complement to the docking scores. In order to estimate the binding energies of tankyrase inhibitors, a preliminary molecular dynamics simulation of 30 ns was performed. The resulting system state was used as a starting point for ten independent runs of 5 ns each as suggested in the work [19]. The mean and confidence interval RMSD (root mean square deviation) values were estimated using the bootstrap procedure for each run and aggregated using mean and L2-norm, respectively.

**Figure 1:**
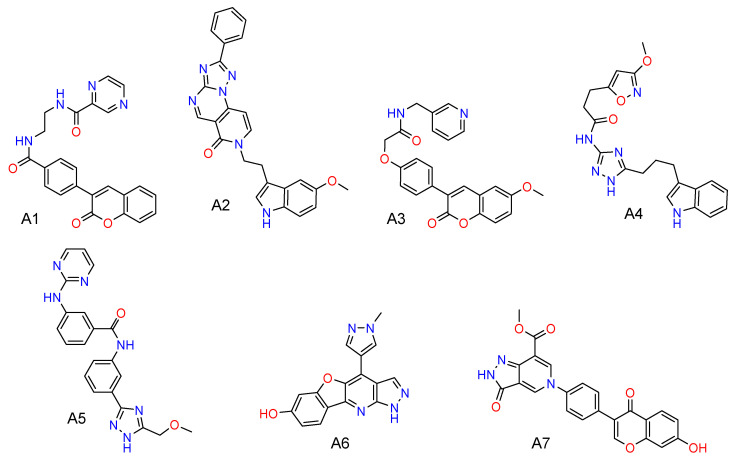
Compounds A1–A7 selected by virtual screening from the subset of the ZINC database.

The molecular docking and the closely related ML-based scoring served as primary screening filters reducing the initial library to the relatively small focused library of 174 compounds. It is worth noting that the distribution of docking scores for the screening library was close to normal with the mean value of −8.5 kcal/mol and the standard deviation of 1.7 kcal/mol. Then the QSAR/QSPR models were used to select 17 compounds for further expert assessment. Seven compounds selected by this virtual screening workflow are shown in Figure 1. These compounds were further evaluated in vitro against the tankyrase enzyme.

### 2.2. Biological Evaluation

**Figure 2:**
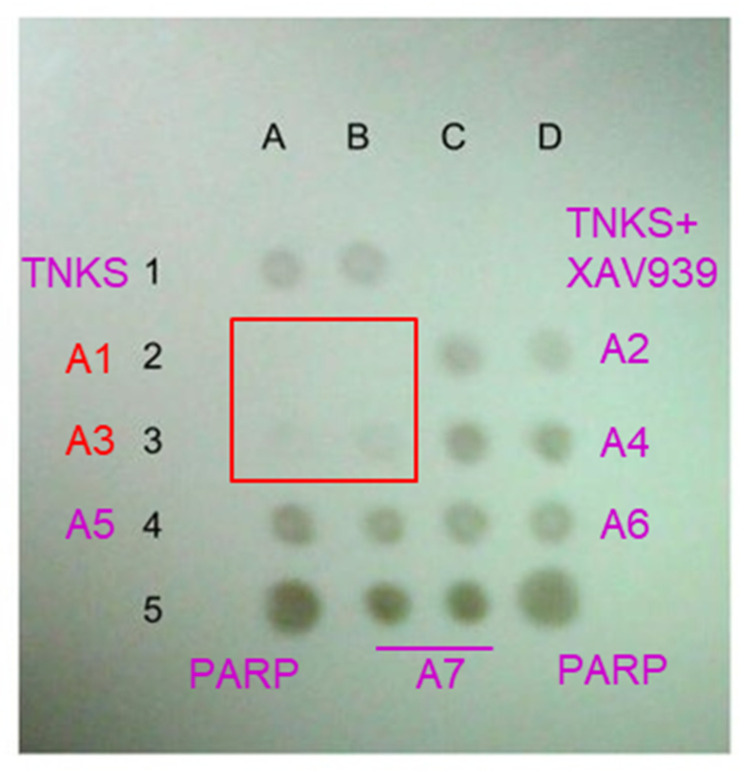
Initial screening results of potential tankyrase inhibitors. Dot blot reflects the amount of the poly-ADP-ribose product of the PARP enzymatic reaction. Positions A1 and B1—tankyrase in the absence of inhibitors; C1 and D1—tankyrase with a positive control inhibitor XAV939, no product; A5 and D5—PARP1 as positive control. Compounds A1–A7 are applied respectively at positions A2 and B2, C2 and D2, A3 and B3, C3 and D3, A4 and B4, C4 and D4, B5 and C5.

The inhibitory activity of the compounds was determined in vitro by measuring the tankyrase enzyme activity using immunochemical assay to detect the accumulation of poly(ADP-ribose) (PAR) in the course of the PARP enzymatic reaction. The initial screening results of the compounds A1–A7 at the concentration of 20 μM and NAD^+^ at 1 μM are shown in Figure 2. It can be seen that PAR is absent only in two positions corresponding to the compound A1. In positions containing the compound A3, the product of the enzymatic reaction is present in a significantly smaller amount than in the absence of inhibition. These data suggest that compounds A1 and A3 likely act as inhibitors of the tankyrase enzyme. These two compounds based on similar scaffolds were selected for further evaluation.

**Figure A1:**
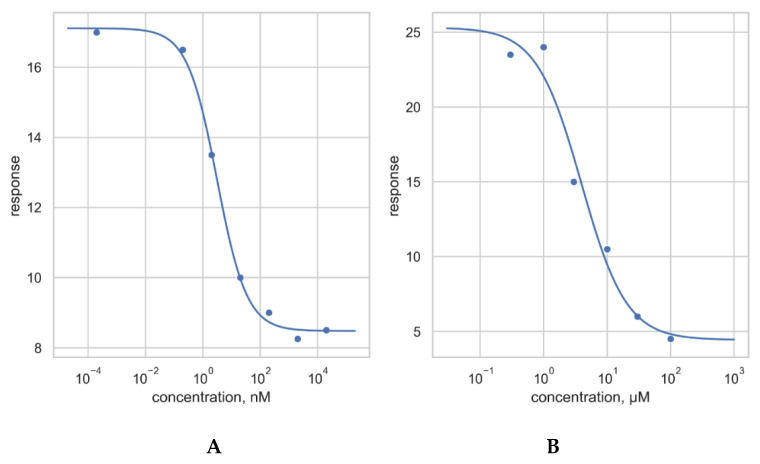
Concentration-response curves for compounds A1 (**A**) and A3 (**B**).

In order to measure their inhibitory activity, the concentration-response curves for the compounds A1 and A3 were obtained at 100 μM NAD^+^ concentration (see Appendix A, Figure A1 and Table A1). Taking into account small number of repeated experiments and high data variance, the IC_50_ values can be cautiously estimated as less than 10 nM for compound A1 and less than 10 μM for compound A3. Moreover, subsequent preliminary experiments suggest that the inhibition for the compound A1 is not competitive and that inhibition of the enzyme by compounds A1 and A3 is independent of the duration of preincubation and is not associated with irreversible inhibition.

### 2.3. Retrospective Analysis of the Virtual Screening Results

Additional studies were performed in order to evaluate to what extent the methods used for the hit-oriented virtual screening could be employed during further lead optimization. In particular, the docking and ML model predictions were recalculated to collect better statistics and compared to the MM-PBSA results.

The molecular docking procedure as implemented in the Autodock Vina and Smina software is stochastic in nature and its different runs result in different ligand positioning in the binding site and, consequently, different scoring function values. To analyze the effect of this uncertainty, one hundred docking runs were performed for each of the A1–A7 compounds starting from various initial conformations and random seeds. The averaged resulting scores and standard deviations are shown in Table 1.

The docking poses obtained on the previous step were also scored using the previously developed target-oriented machine learning scoring approach [13]. The model employed in the present study was refined taking into account additional data available in the most recent version of the ChEMBL 26 database [20]. Similar to the built-in scoring functions, we collected the predicted activity probabilities and their standard deviation values (see Table 1).

As can be seen from Table 1, for all the compounds previously selected for the in vitro testing, the docking scores and MM-PBSA energies are quite similar and promising. The binding probabilities predicted by the ML model show a greater variance—the model predicts that the compounds A1, A2, A3, A6 and A7 could be binders with decent probability while the probabilities for the A4 and A5 compounds are relatively lower. However, taking into consideration the results from all three components of the consensus scoring approach, the data would suggest significant likelihood of binding for all the compounds.

### 2.4. Molecular Dynamics Studies

Looking retrospectively at the values presented in Table 1, one can see that almost all the predictions are quite similar although only two compounds have shown some level of inhibitory activity in the experiment. Obviously, such scoring techniques cannot be used to predict the effect of structural modification on the binding affinity or to rank the compounds based on their binding energies.

A predictive model capable of suggesting and evaluating such modifications would be highly useful and desirable in the subsequent lead optimization stages. Thus, the binding of the discovered active compounds was analyzed in more detail to get quantitative insight into the relations between the estimated binding energy and the inhibitory activity level. Then the binding energies were estimated via the free energy perturbation (FEP) method that is believed to be more accurate than molecular docking or MM-PBSA method.

#### 2.4.1. Binding Modes

**Figure 3:**
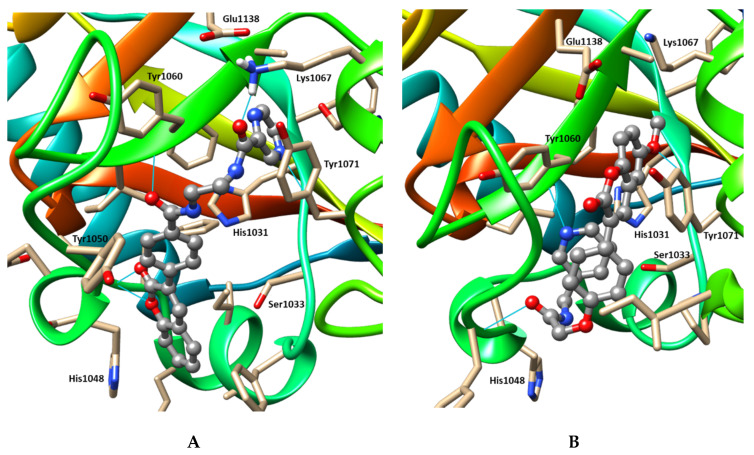
Binding modes of compounds A1 (**A**) and A3 (**B**) predicted by molecular docking and molecular dynamics. The ligand molecule is represented by grey ball-and-stick model. The amino acid residues located within 4 Å from it are shown as beige stick models. Hydrogen bonds are shown by cyan lines.

The binding modes of compounds A1 and A3 predicted by molecular docking and refined by molecular dynamics are shown in Figure 3. As can be seen, despite the scaffold similarity, A1 and A3 have been predicted to bind differently. Although localized roughly in the same pocket, the compounds take different conformations with apparent intramolecular π-π stacking in A3. Thus, compound A1 is expected to form hydrogen bonds with the backbone atoms of Gly1032, Asp1045, Tyr1060 and the side chain of Lys1067 and interact with His1048 through π-π stacking. The compound A3 could interact with Tyr1060 and Tyr1071 via π-π stacking and form hydrogen bonds with the backbone atoms of Gly1032, Tyr1050 and Tyr1060.

**Figure 4:**
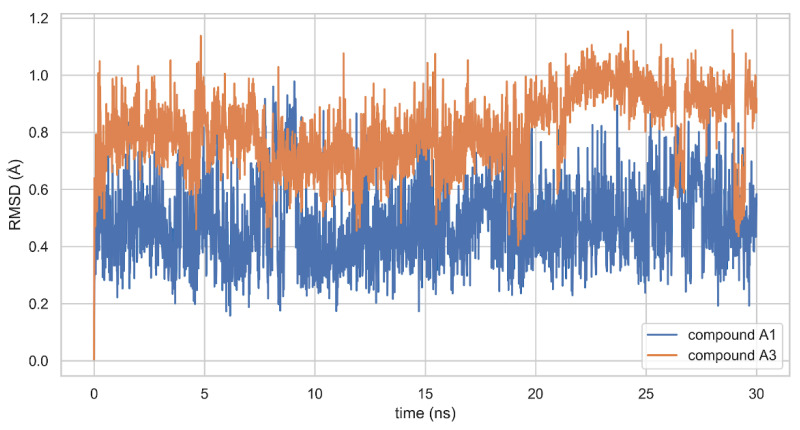
RMSD values from the initial coordinates for compounds A1 and A3 in the protein-ligand complexes.

The molecular dynamics simulations suggest that these binding modes are stable. At least on the time scale of 30 ns, the RMSD values for ligands are fluctuating around small constant value (less than 1 Å) and do not show any distinctive trends (see Figure 4).

#### 2.4.2. FEP Calculations

The alchemical free energy perturbation (FEP) method is based on a non-physical thermodynamic cycle where the binding free energy is computed as the sum of multiple steps involving ligand coupling/decoupling in the bound and unbound states. This method could be seen as an enhanced sampling approach as it allows one to preserve the binding mode of the decoupled ligand. It is broadly acknowledged as more robust compared to molecular docking and overall is in the best agreement with the experiment [21].

The energy differences calculated for each stage of the alchemical thermodynamic cycle (see Section 3.5.3 for detailed explanation) are listed in Table 2. The absolute binding energies for compounds A1 and A3 having very similar scaffolds were estimated as −10.8 ± 0.2 kcal/mol and −4.0 ± 0.2 kcal/mol respectively. These correspond to the p*K_d_* values of 7.9 and 2.9, in reasonable agreement with the approximate experimental pIC_50_ values of 8.0 and 5.0 for compounds A1 and A3, respectively.

These results suggest a better explanation of the observed difference in the inhibitory activity of the compounds A1 and A3 compared to the MM-PBSA method. According to the calculated FEP values, the free energy of decoupling and restraining the ligand in a complex is the major source of the overall differences in binding energies. In contrast to the docking results, these estimates suggest that compound A1 likely has more favorable interactions in the binding site compared to compound A3 which can be attributed to the differences in the binding modes and the presence of additional polar groups. The energy differences for the decoupling from solvent are dominated by the Coulomb term and have an opposite effect on the total binding energy (probably also attributable to additional polar interactions for A1) but their absolute size is significantly smaller. Finally, the difference in the restraint energies is negligible, apparently reflecting similar conformational flexibility of the compounds.

As an additional check of the FEP applicability to compounds ranking, we also performed the calculations for compound A2 and A7. Despite their good scores estimated by molecular docking, ML model and MM-PBSA approaches, the compound A2 shows only very weak inhibitory activity while for the compound A7 no inhibition was detected. The resulting free energy values are listed in Table 2. The total free energy of binding can be estimated as −8.2 ± 0.2 kcal/mol for compound A2 and as 7.6 ± 0.2 kcal/mol for compound A7. Surprisingly, the calculated binding energy for the virtually inactive compound A2 appears to be better than for the discovered inhibitor A3. This issue is most probably explained by the insufficient sampling during the FEP calculations, which could in principle be addressed by extension of the simulation. The observed inhibitory activity may also be affected by other processes besides the ligand-target binding. Nevertheless, even with such discrepancy, the binding energies predicted by FEP are in better agreement with the experiment compared to the ones provided by the previously applied methods, since they correctly rank compound A1 as much more potent than either A2 or A3. On the other hand, although the compound A7 shares substantial scaffold and structure similarity with compounds A1 and A3, its total free energy of binding was predicted to be positive indicating unfavorable thermodynamics of binding, primarily attributable to the ligand decoupling in solution. This is also in good agreement with the experimental inhibition data.

Overall, the predictions based on FEP calculations are not quite perfect but more reliable compared to the docking and MM-PBSA methods. That could be related to the explicit accounting for the solvation effects, in contrast to the docking-based methods or continuum solvation models used by the MM-PBSA approach. These preliminary results suggest that the FEP-calculated binding energies could potentially be used to guide lead optimization of novel TNKS inhibitors. However, a more detailed and thorough study is required in order to choose optimal parameters of the simulation. Also, it is highly desirable to conduct a separate study to evaluate the correlation of FEP energies with available experimental activity values of the known inhibitors. Alternatively, the novel machine learning approach to the analysis of the molecular dynamics trajectories [22] could be applied to predict the inhibitor activities.

## 3. Materials and Methods

### 3.1. Virtual Screening Library

The library of compounds for virtual screening was formed based on the ZINC 15 database [17]. Non-reactive compounds (*anodyne* reactivity as defined in the database) were selected from the predefined drug-like subset. These compounds were further filtered with respect to potential suppliers in order to ensure their availability for ordering in our region. The ChemAxon Instant JChem 19.23 software package (https://chemaxon.com, ChemAxon, Budapest, Hungary) was used for structure database management. The 3D coordinates of the downloaded chemical structures were generated from the SMILES format using RDKit version 2019.03.4 Python library (http://www.rdkit.org). The resulting set of the compounds included about 1.7M distinct chemical structures.

### 3.2. Molecular Docking

Based on our previous study [13], the structure of the TNKS2-ligand complex (PDB: 4N4V) was chosen for optimal performance of molecular docking-based virtual screening. Semi-rigid docking procedure was performed by means of Smina version 2019.10 software [23]. The search box was centered on the bound ligand and extended by default offset of 10 Å from the ligand in every direction. The *Vinardo* scoring function was used to find a binding pose and the exhaustiveness parameter was increased to 20 from the default value of 8. The compounds having the scoring function value below −12 kcal/mol were selected.

### 3.3. Prediction of Physicochemical and ADMET Properties

The assessment of a number of key physicochemical properties, pharmacokinetic parameters and toxicity endpoints was carried out for the compounds that passed the initial docking-based filter. The following predicted properties were considered as acceptable at this early stage of the search for lead compounds—molecular weight MW < 600; lipophilicity LogP < 6 (estimated using our previously developed model and the ALogPS 3.0 model available on the OCHEM (online chemical modeling environment) platform [24]); aqueous solubility > 10^−5^ M (estimated using the ALogPS 3.0 model available on the OCHEM platform [24]); human intestinal absorption HIA > 75% (estimated using our previously developed model [25]); low risk of hERG-mediated cardiac toxicity (channel affinity p*K_i_* < 6 and inhibitory activity pIC_50_ < 6 as estimated using our previously developed models [26]). The blood-brain barrier permeability (LogBB) values were also estimated using our previously developed model [27] to provide additional data for expert evaluation and selection of the compounds. The predictive HIA, hERG and LogBB models based on accurate and representative training sets, fragmental descriptors and artificial neural networks are available in the integrated online service for ADMET (absorption, distribution, metabolism, excretion, toxicity) properties prediction (ADMET Prediction Service, http://qsar.chem.msu.ru/admet/). The predicted properties for the selected compounds are listed in the Appendix B (Table A2).

### 3.4. Biological Evaluation

The tankyrase enzyme activity was determined in vitro using the immunochemical assay based on measuring the accumulation of poly(ADP-ribose) (PAR) in the course of the PARP enzymatic reaction. PAR sorbed on nitrocellulose binds murine anti-PAR antibodies that, in turn, bind to peroxidase-conjugated antibodies against murine immunoglobulins. Peroxidase activity is determined by the chemiluminescent method via luminol oxidation, with the resulting luminescent product illuminating the X-ray film.

The tankyrase 2 catalytic fragment (human, recombinant, residues 849–1166) and its known inhibitor XAV939 were purchased from Sigma-Aldrich (St. Louis, MO, USA). Poly(ADP-ribose) polymerase 1 (PARP1) from the HT F Homogeneous PARP Inhibition Assay Kit (Amsbio) was used as a positive control. The control inhibitor or the test compounds (InterBioScreen, Moscow, Russia) were dissolved in dimethyl sulfoxide (DMSO). An enzyme was incubated in the presence or absence of the compounds in a reaction mixture comprising 100 mM Tris-HCl (pH 8), 2 mM MgCl_2_, 2% DMSO and NAD^+^ for 30 min at 25 °C. The total volume of the mixture was 5 μL. Upon completion of the enzymatic reactions, the reaction mixture was applied in duplicates as dots on nitrocellulose matrix (Protran Nitrocellulose Transfer Membrane BA85, Whatman) that was then successively treated with the bovine serum albumin (BSA) in 0.9% NaCl and the Anti-PAR (Ab-1) Mouse mAb (10H) (Sigma-Aldrich) diluted 1:600 in the same solution. The antigen-antibody reaction was carried out for at least 1 h at room temperature or overnight at 4 °C. Next, the primary antibodies were washed off with the TBST buffer (10 mM Tris-HCl, 0.9% NaCl, 0.1% Tween 20, pH 7.4) and nitrocellulose was treated with the peroxidase-conjugated goat anti-mouse IgG antibodies (Fab specific) (Sigma-Aldrich) diluted 1:60,000. After one-hour incubation, the secondary antibodies were also washed off with TBST, nitrocellulose matrix was transferred to the solution of SuperSignal West Pico Chemiluminescent Substrate (Thermo Scientific) and exposed to an X-ray film for signal detection. The signal was quantified using ImageJ 1.52a software [28].

The IC_50_ values were estimated from the concentration-response curves using the in-house Python script based on the SciPy library 1.4.1 (*scipy.optimize.curve_fit* function) by fitting the four parameter logistic (4PL) regression model [29]:

$$Y=MIN+\frac{MAX-MIN}{1+{\left(\frac{C}{IC_{50}}\right)}^{H}}$$

where Y is the measured response, C is the inhibitor concentration, MIN and MAX are the minimum and maximum observed response values, $IC_{50}$ is the half-maximal inhibitory concentration and H is the Hill coefficient. The model parameters are listed in the Appendix A (Table A1).

### 3.5. Molecular Dynamics Studies

#### 3.5.1. Basic Molecular Dynamics Simulation Protocol

Initial structures of the tankyrase complexes with inhibitors were obtained by molecular docking of the compounds into the tankyrase-2 enzyme (PDB: 4TK5). The missing residues in the experimental protein structure were reconstructed by homology modeling using the MODELLER v.9.19 program [30] and the unresolved amino acid side chains were modeled using the Dunbrack rotamer library [31]. The atomic charges for the ligand molecules were calculated by the AM1-BCC method in the *antechamber* program from the AmberTools 16 package [32]. The ligand parameters were modeled using the General Amber Force Field (GAFF) [33]. Amber topology and coordinate files using explicit solvent (TIP3P water model, 0.15M NaCl) and dodecahedron periodic box with 10 Å margin were created by the *parmchk* and *tleap* programs from the AmberTools package using the AMBER99SB force field [34]. The data files were converted to the GROMACS format using the *acpype* script [35]. All subsequent molecular dynamics simulations were performed using GROMACS 2019.4 [36] software. After the initial relaxation by the steepest descent minimization algorithm, the system was subjected to temperature stabilization (300 K) in the NVT ensemble for 100 ps (50,000 steps of 2 fs each) using the modified Berendsen thermostat. Then the molecular dynamics simulation was performed in the NPT ensemble for 100 ps (50,000 steps of 2 fs each) using the Berendsen barostat in order to stabilize the pressure at 1 atm. After the temperature and pressure were equalized, a production molecular dynamics simulation was performed using v-rescale thermostat and Parrinello-Rahman barostat on the NVIDIA GTX 1080Ti high-performance GPU.

#### 3.5.2. MM-PBSA Calculations

In order to estimate the binding energies of the tankyrase-inhibitor complexes, a preliminary molecular dynamics simulation of 30 ns was performed according to the basic protocol described above. The resulting system state was used as a starting point for ten independent runs of 5 ns each as suggested in the work [19]. The bootstrap procedure [37] was used to estimate the mean and confidence interval RMSD values of the calculated binding energies for each run that were then aggregated using mean and L2-norm, respectively.

The binding energy was calculated by the single-trajectory MM-PBSA method using the *g_mmpbsa* standalone software [37]. To reduce the computational cost, 251 equidistant frames from a time interval of 1–5 ns in the 5 ns simulation trajectory were used. The solute dielectric constant was set to 4. The binding free energy was estimated as:

$$\Delta G_{bind,aq}=\Delta H-T\Delta S\approx \Delta E_{MM}+\Delta G_{solv}-T\Delta S$$

where $\Delta G_{bind,aq}$ is the binding free energy, $\Delta E_{MM}$ is the molecular mechanics energy change consisting of the electrostatic energy change and the van der Waals energy change, $\Delta G_{solv}$ is the solvation free energy change consisting of polar and non-polar terms (in this study, the Poisson-Boltzmann solver was used for the polar term and the SASA model for non-polar contribution) and TΔS is the entropy term. Due to high computational demand, the entropy term was omitted during this study (moreover, a number of works suggest that its inclusion does not lead to any improvement in the ranking of relative binding affinities [38,39]).

#### 3.5.3. Absolute FEP Calculations

The alchemical free energy perturbation (FEP) method is based on a non-physical thermodynamic cycle comprising the following states—(1) physical unbound state, (2) alchemical state where the ligand is decoupled from the solution, (3) alchemical state where the ligand is decoupled and restrained in the binding site, (4) physical bound state. This method could be seen as an enhanced sampling approach as it allows to preserve the binding mode of the decoupled ligand. The binding free energy is calculated as:

$$\Delta G_{bind}^{0}=-\Delta G_{elec+vdw+restr}^{prot}+\Delta G_{elec+vdw}^{solv}+\Delta G_{restr}^{solv}$$

where $\Delta G_{bind}^{0}$ is the free energy of binding, $\Delta G_{elec+vdw+restr}^{prot}$ is the free energy of decoupling and restraining the ligand in a complex, $\Delta G_{elec+vdw}^{solv}$ is the free energy of decoupling the ligand in solution and $\Delta G_{restr}^{solv}$ is the free energy for restraining the decoupled ligand in solution (due to the closed-form analytical expression, it demands very little computational overhead).

The initial states of the tankyrase-inhibitor complexes for the FEP calculations were obtained from the same preliminary 30 ns molecular dynamics simulations that were used for the MM-PBSA calculations. Absolute binding free energy calculations were performed using standard GROMACS tools according to the methodology [40]. The free energy differences across the intermediate states were calculated using the Multistate Bennett Acceptance Ratio (MBAR) approach [41] implemented in the *alchemical_analysis.py* Python script [42].

Ligand decoupling from a complex involves gradually turning off the Coulomb and Lennard-Jones interactions as well as restraints over a total of 30 windows controlled by linearly spaced lambda values. For each lambda value, the energy minimization, NVT and NPT equilibration and 1 ns production run were performed, followed by the MBAR-based estimation of the free energy. Similarly, ligand decoupling from solvent comprises a total of 20 windows with linearly spaced lambda values (in this case, only Coulomb and Lennard-Jones interactions were turned off). The length of the production runs for this step was set to 5 ns. For consistency reasons, the MBAR free energy estimation was also performed. The set of the binding site restraints was defined by one distance, two angle and three dihedral harmonic potentials according to work by Boresch et al. [43].

## 4. Conclusions

In the course of this study, the virtual screening of more than 1.7 million commercially available compounds was performed based on the molecular docking results, predicted physico-chemical and ADMET properties and molecular dynamics simulations. In the in vitro biological evaluation, two out of seven selected compounds showed a decent level of inhibitory activity against the TNKS2 enzyme with the IC_50_ values of less than 10 nM and 10 μM.

The in-depth retrospective analysis revealed that the molecular docking-based scoring functions or MM-PBSA calculations are not suitable for accurate ranking the compounds according to their affinity values and can only be used for binary classification to select potential hits. In contrast, an alternative approach based on the free energy perturbation (FEP) calculations, although not perfect, allowed us to establish a more reliable correlation between the binding energy and inhibitory activity of four compounds and to explain some differences in their activity in spite of very similar scaffolds. That could be related to the explicit accounting for the solvation effects, in contrast to the docking-based methods or continuum solvation models used by the MM-PBSA approach.

Thus, instead of the fast and cheap docking-based scoring, we propose the free energy calculations as a possible method for the subsequent lead optimization stages. However, it should be applied only after additional detailed study aiming to select optimal parameters and evaluate its ranking performance on a large set of known inhibitors.

## Figures and Tables

**Table 1:** Predicted binding affinities/scores and standard deviations for compounds A1–A7.

Compound	Binding Affinity Predicted by Docking Scoring Function, kcal/mol	Binding Probability Predicted by ML Scoring Function	Binding Energy Calculated by MM-PBSA, kcal/mol
A1	−12.8 ± 0.1	0.61 ± 0.1	−32.5 ± 10.3
A2	−12.4 ± 0.2	0.70 ± 0.1	−36.3 ± 9.8
A3	−12.4 ± 0.1	0.62 ± 0.1	−30.8 ± 9.2
A4	−11.7 ± 0.1	0.24 ± 0.1	−28.1 ± 9.6
A5	−12.6 ± 0.2	0.15 ± 0.1	−29.1 ± 9.7
A6	−12.5 ± 0.1	0.46 ± 0.1	−31.2 ± 8.0
A7	−12.6 ± 0.1	0.56 ± 0.1	−32.0 ± 8.8

**Table 2:** Free energy differences calculated for each stage of the alchemical thermodynamic cycle for compounds A1, A2, A3, A7.

Binding Free Energy (kcal/mol)		A1	A2 ^a^	A3 ^a^	A7 ^a^
**Free energy of decoupling and restraining the ligand in a complex**	$-\Delta G_{elec+vdw+restr}^{prot}$	**−49.5 ± 0.2**	** −35.2 ± 0.2 (14.3)**	** −32.9 ± 0.2 (16.6)**	** −39.4 ± 0.2 (10.1)**
Coulomb term	$-\Delta G_{elec}^{prot}$	−30.2 ±0.1	−15.9 ± 0.1 (14.3)	−20.6 ± 0.1 (9.6)	−22.1 ± 0.1 (8.1)
van der Waals andrestraint term	$-\Delta G_{vdw+restr}^{prot}$	−19.3 ± 0.1	−19.3 ± 0.2 (0.0)	−12.3 ± 0.1 (7.0)	−17.3 ± 0.1 (2.0)
**Free energy of decoupling the ligand in solution**	$\Delta G_{elec+vdw}^{solv}$	** 31.4 ± 0.1**	** 20.3 ± 0.1 (−11.1) **	** 22.0 ± 0.1 (−9.4) **	** 40.2 ± 0.1 (8.8) **
Coulomb term	$\Delta G_{elec}^{solv}$	29.5 ± 0.1	18.1 ± 0.1 (−11.4)	21.5 ± 0.1 (−8.0)	37.1 ± 0.1 (7.6)
van der Waals term	$\Delta G_{vdw}^{solv}$	1.9 ± 0.1	2.2 ± 0.1 (0.3)	0.5 ± 0.1 (−1.4)	3.1 ± 0.1 (1.2)
**Free energy for restraining the decoupled ligand in solution**	$\Delta G_{restr}^{solv}$	** 7.3 **	** 6.7 (−0.6)**	** 7.0 (−0.3)**	** 6.8 (−0.5)**
**Total free energy of binding**	$\Delta G_{bind}^{0}$	**−10.8 ± 0.2**	**−8.2 ± 0.2 (2.6)**	**−4.0 ± 0.2 (6.8)**	**7.6 ± 0.2 (18.4)**

**Note:**^a^ΔG values relative to the compound A1 are listed in parentheses.

## Appendix A. Concentration-Response Curves for Compounds A1 and A3

The concentration-response curves for compounds A1 and A3 are shown in Figure A1. Measurements were carried out at 100 μM NAD^+^ concentration according to the procedure described in Materials and Methods (Section 3.4). The response values are the means of duplicate points.

The IC_50_ values were estimated using in-house Python script based on the SciPy library 1.4.1 (*scipy.optimize.curve_fit* function) by fitting the four-parameter logistic (4PL) regression model:

$$Y=MIN+\frac{MAX-MIN}{1+{\left(\frac{C}{IC_{50}}\right)}^{H}}$$

where Y is the measured response, C is the inhibitor concentration, MIN and MAX are the minimum and maximum observed response values, $IC_{50}$ is the half-maximal inhibitory concentration and H is the Hill coefficient. The model parameters are listed in Table A1. Taking into account small number of repeated experiments and high data variance, the IC_50_ values can be cautiously estimated as less than 10 nM for compound A1 and less than 10 μM for compound A3.

**Table A1:** Parameters of the Concentration-Response Curves for Compounds A1 and A3.

Compound	MIN	MAX	$IC_{50}$	H
A1	8.5 ± 0.2	17.1 ± 0.2	3.1 ± 0.5 nM	0.8 ± 0.1
A3	4 ± 2	25 ± 3	4 ± 2 μM	1.2 ± 0.6

## Appendix B. Prediction of Physicochemical and ADMET Properties of Compounds A1–A7

The prediction of key physicochemical and ADMET properties during virtual screening was performed using the methods and procedure described in Materials and Methods (Section 3.3). The predicted values for compounds A1–A7 selected by virtual screening are listed in Table A2.

**Table A2:** Predicted Physicochemical and ADMET Profiles of Compounds Selected by Virtual Screening.

Compound	MW	LogPow	pS	LogBB	HIA	hERG p*K_i_*	hERG pIC_50_
A1	414.42	1.98	4.35	0.53	100.0	4.26	4.00
A2	436.48	2.57	4.72	−0.60	100.0	5.04	5.03
A3	416.43	3.33	4.94	−0.46	90.8	5.65	4.50
A4	394.44	2.76	4.23	−1.23	100.0	5.49	5.63
A5	401.43	3.10	4.05	1.52	93.0	4.86	4.65
A6	305.30	2.38	3.71	0.20	87.5	4.04	4.66
A7	429.39	2.23	4.42	−1.10	97.6	5.05	5.92

**Note:** MW—molecular weight, LogPow—octanol-water partition coefficient, pS—aqueous solubility [−log(M)], LogBB—blood-brain barrier permeability, HIA—human intestinal absorption [%], hERG *pK_i_*—hERG potassium channel affinity [−log(M)], hERG pIC_50_—hERG potassium channel inhibitory activity [−log(M)].
